# Dr. Edwin E. Whitcomb

**Published:** 1901-09

**Authors:** 


Dr. Edwin E. Whitcomb, of Rochester, N. Y., U. of B., '75, died at his home August 1, 1901, aged 50 years.

